# Evaluation of Wood and Cellulosic Materials as Fillers in Artificial Diets for *Lyctus africanus* Lesne (Coleoptera: Bostrichidae)

**DOI:** 10.3390/insects6030696

**Published:** 2015-07-23

**Authors:** Titik Kartika, Tsuyoshi Yoshimura

**Affiliations:** 1 Research Institute for Sustainable Humanosphere, Kyoto University, Uji Campus, Kyoto 6110011, Japan; E-Mail: tsuyoshi@rish.kyoto-u.ac.jp; 2 Research Centre for Biomaterials, Indonesian Institute of Sciences, Jl. Raya Bogor Km. 46, Cibinong 16911, Indonesia

**Keywords:** artificial diet, filler, cellulose, growth, *L. africanus*

## Abstract

We studied the usefulness of wood- and cellulose-based diets for *L. africanus* Lesne. Three diets were prepared which differed on the base ingredients; wood particles (Diet 1), cellulose powder (Diet 2), and alpha-cellulose (Diet 3). The diets were provided to adult *L. africanus* and the number of larvae, as well as the number of adults that emerged sex ratio, and body weight of the progeny was determined. Findings indicated similar results for the number of larvae, sex ratio and body weight of the emerged *L. africanus* fed on each diet. However, the number of adult produced by *L. africanus* on Diet 3 was significantly lower. The results indicate that the amount of vital nutrients is not the only important factor in selecting a suitable diet for *L. africanus* because the filler used in artificial diets influences the beetles overall population growth. For the population upon which the diets were tested, Diet 1 and Diet 2 could be utilized to rear beetles in the laboratory.

## 1. Introduction

There are a number of beetles from the family Bostrichidae subfamily Lyctinae that are considered indigenous to tropical and temperate regions [1,2,3]. Several species of the Lyctinae, [4], have been introduced into Australia [5,6,7], New Zealand [8,9], USA/Canada, Europe, countries around the Mediterranean [1,2,3,10] and Japan [2,11,12]. Several species of genus *Lyctus* are reported to be distributed in all faunal regions, but are supposed to be an endemic to the Oriental and Ethiopian regions [1].

In Japan, *L. africanus* along with *L. brunneus* have frequently been found in some prefectures [12]. These two species are morphologically quite similar to each other [1]. *Lyctus brunneus* has been extensively studied because of its widespread distribution as a pest of timber and wood products [6,13]. However, *L. africanus* is also found to infest timber and timber products, including plywood [1,2], dried roots, seeds and tubers [1,14] and incidence of *L. africanus* infestations is increasing in Japan [12].

The *Lyctus* are often referred to as powder post beetles because the larvae tunnel through and feed on wood, producing frass that resembles a fine powder. It is the larvae that cause damage to sawn hardwood timber and manufactured wood products. Laboratory studies of *Lyctus* have typically been conducted using an artificial diet instead of wood. *Lyctus* require starch, protein and other vital nutrients [15,16] therefore, artificial diets used in *Lyctus* studies have consisted mostly of starch and protein supported with a filler to enhance the physical properties of the diets. Most of the fillers used in the artificial diets have been from the sapwood portion of hardwood species, such as *Shorea* sp., in particular combination with other nutrients to support growth [16,17].

The combination of either *Shorea* sp*.* sawdust or cellulose powder blended with other nutrients was proposed as an ideal diet for culturing, *L. brunneus* [16]*.* In 1962, a study on *L. africanus* reported that this beetle was successfully bred on an artificial diet consisting of a mixture containing 90 parts wheat flour and 10 parts yeast [14]. Unfortunately, there was no information on the size of the adult beetle population produced with this artificial diet. Therefore, we evaluated the usefulness of several artificial diets, including the ideal diet for *L. brunneus*, to examine growth of *L. africanus.*

The use of wood as a filler may be problematic for the mass culturing of *Lyctus* beetles in light of its availability, time-consuming preparation, and the varying distribution of nutrients in different pieces of wood, even within the same log. In contrast, cellulosic materials are commercially available. Here, we discuss and determine the utility of wood- and cellulose-based diets for *L. africanus* by examining some parameters related to insect fecundity. In addition, by clarifying the favorable diet conditions for *L. africanus*, we discuss the possibility of obtaining greater numbers of adults in laboratory settings for further study.

## 2. Experimental Section

### 2.1. Insect Sources

Adult-stage *L. africanus* bred in a laboratory culture were used for this study. The culture had been maintained on an artificial wood-based diet contained sawdust, starch and yeast for about 20 years [18] in the Laboratory of Innovative Humano-habitability, Kyoto University, Japan. The culture was maintained in a dark climatic chamber at 26 °C and 65% relative humidity (RH).

### 2.2. Diet Sources

Starch, protein and filler were the main components of the three artificial diets. The diets were differed according to the type of filler, as follows: Diet 1 contained the sapwood portion of *Shorea* sp. ground and sieved through a 20–40 mesh screen and two kinds of commercially available cellulose materials Diet 2 contained cellulose powder (Nacalai Tesque, Kyoto, Japan) and Diet 3 alpha-cellulose powder (Nacalai Tesque). The alpha-cellulose powder was considered true cellulose due to its high purity [18]. Vital nutrients were provided to each of the three diets by adding soluble starch (Nacalai Tesque) and dried brewer’s yeast powder (Asahi Food and Health Care, Tokyo, Japan). Diets 1 and 2 were the common artificial diets for *L. brunneus* [16], whereas Diet 3 was an additional diet tested for *L. africanus* in the present study.

### 2.3. Diet Preparation

Diets were prepared by mixing the components (Table 1) with distilled water, as follows: 70% for the wood-based Diet 1 and 90% the two cellulose-based diets (Diet 2 and Diet 3). Basically, each diet was mixed with water and made into dough that was compacted into brick-shaped blocks. In this study, two different sized blocks were used; 1) for the larval stage observation (larval number), 12 g of a mixed diet was prepared into 2 × 2 × 2 cm bricks and 2) for the adult stage observation (total population, sex ratio, and body weight), 100 g was pressed into 8 × 4 × 2 cm bricks. The pressed diet blocks were oven-dried at 60 °C for 3 days and stored in a plastic box at 26 °C and 65% relative humidity in the dark until used in the test.

**Table 1:** Composition of the three artificial diets for *L. africanus.*

Composition	Diet 1 (%, *w/w*)	Diet 2 (%, *w/w*)	Diet 3 (%, *w/w*)
Wood powder	26	−	−
Cellulose powder	−	26	−
Alpha-cellulose	−	−	26
Dry yeast	24	24	24
Starch	50	50	50

Note: on w/w is a mass percentage of each diet component in a total mass of diet mixture.

### 2.4. Total Larvae

Two blocks of the diet were placed in a closed Petri dish (125-mm dia., 30 mm height) covered with filter paper (125-mm dia., Whatman No. 2, GE Healthcare, UK) and maintained in an environmental chamber at 26°C and 65% relative humidity (RH). Five pairs of newly emerged male and female adults *L. africanus* were introduced into each dish. The number of larvae in the dish was counted after 45–50 days using the flotation method. The artificial diet was immersed in water and the floating larvae counted to estimate the oviposition potential of the adult *L. africanus* beetles originally placed in the petri dish. This experiment was replicated six times.

### 2.5. Population, Sex Ratio and Body Weight of Newly Emerged Adults

Two blocks of the diet (8 × 4 × 2 cm) were put in a glass jar (450 mL with 65 mm dia., 125 mm height) with a filter paper (70-mm dia., Whatman No. 2, GE Healthcare, UK) on the bottom. Five pairs of newly emerged male and female *L. africanus* were introduced into each jar and allowed to oviposit. The jar was capped with a filter paper (70-mm dia., Whatman No. 2, GE Healthcare) to permit ventilation. The jar was stored in a dark climatic chamber (26 °C, 65% RH) until new adults emerged. Eight replications of each experimental unit were completed.

Emerged adults were observed and total population, sex ratio, and body weight evaluated. The observations were conducted at two-day intervals throughout the emergence period. The sex of each beetle was determined by the presence or absence of a heavy fringe of hair along the hind margin of the terminal abdominal sternite [1]. Body weight was measured by weighing all of the harvested adults and estimated by calculating average body weight using the number of adults. The data were analyzed by one-way analysis of variance (ANOVA), with the Tukey-Kramer HSD test at 5% critical difference as a post-hoc test.

## 3. Results

### 3.1. Total Larvae (Immature-Stage)

The average number of larvae in a dish 141.00 for Diet 1, 134.17 for Diet 2, and 113.83 for Diet 3 was not significantly different among the three diets (*F* = 0.45, *df = * 2, *P* = 0.65) (Table 2).

**Table 2:** Average number of larvae *L. africanus* in three artificial diets.

Artificial diet	Total larvae/5 female (mean ± S.E)
Diet 1	141.00 ± 10.41 ^a*^
Diet 2	134.17 ± 24.48 ^a^
Diet 3	113.83 ± 25.00 ^a^

Note: * Same letter is not significantly different (Tukey-Kramer HSD test; *p* < 0.05) following one-way ANOVA.

### 3.2. Total Adults

**Figure 1:**
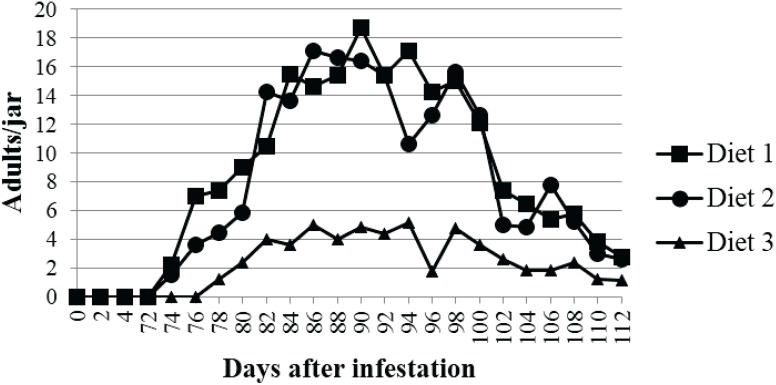
Average number of newly emerged adults of *L. africanus* fed different diets (Diet 1: wood particle-based diet; Diet 2: cellulose-powder based diet; Diet 3: α-cellulose-based diet).

The first adult emergence occurred after 10 weeks (Figure 1). New adult beetles emerged from Diet 1 and Diet 2 five days earlier than from Diet 3. The number of emerging beetles increased in the first two weeks, and then slowly decreased by the sixth week. The total populations from Diet 1 and Diet 2 were significantly bigger than Diet 3 (*F = * 5.57, *df = * 2, *p* = 0.011). Diet 1 and Diet 2 generated 206 and 188.87 total adults, on average, respectively, while Diet 3 produced 55.87.

### 3.3. Sex Ratio

The adult females tended to be produced in higher numbers than adult males in every diet (Table 3).

### 3.4. Body Weight

The three diets did not affect the body weight of newly emerged adults of *L. africanus* (*F* = 0.32, *df =* 2, *p* = 0.731). The average body weight of *L. africanus* fed on Diet 1, Diet 2 and Diet 3 were 1.98 ± 0.04 mg, 1.87 ± 0.06 mg, and 2.01 ± 0.21 mg, respectively (Table 3).

**Table 3:** Development of *L. africanus* in three artificial diets.

Artificial diet	Total adults	Sex ratio (M/F)	Body weight (mg)
Diet 1	206.00 ± 49.37 ^a*^	0.94 ± 0.06 ^a^	1.98 ± 0.04 ^a^
Diet 2	188.87 ± 30.60 ^a^	0.80 ± 0.05 ^a^	1.87 ± 0.06 ^a^
Diet 3	55.87 ± 16.18 ^b^	0.76 ± 0.08 ^a^	2.01 ± 0.21 ^a^

Note: * Same letter within a column is not significantly different (Tukey-Kramer HSD test; *P* < 0.05) following one-way ANOVA.

## 4. Discussions ad Conclusions

All three diets allowed *L. africanus* to complete their entire life cycle. The period from oviposition to adult emergence in this study were similar to those in a previous study [14]. However, a significantly higher population of adults was obtained on Diet 1 and Diet 2 than Diet 3, although the fecundity, as measured by larval numbers at 45 days, did not differ among the diets. It has been reported that wood-based and cellulose-based diets (Diet 1 and Diet 2) improved the growth of *L. brunneus* [16,19]. In terms of easy handling and time-saving preparation the commercial sources of cellulose when used as a raw material for mass rearing, is more suitable for *L. brunneus* and *L. africanus* than a diet of wood particles (sawdust) [19]. The chemical grade cellulose powder contains a small amount of hemicellulose and other substances [18] which *L. brunneus* might have utilized the polysaccharides intermediate in composition between starch and the hemicelluloses [20]. In contrast, Diet 3 did not improve the growth of this *Lyctus*. The physical and/or chemical properties of alpha-cellulose fiber in Diet 3 might impede the development of adults due to an attribute of alpha-cellulose fiber that contains anhydroglucose chains [18].

Most studies on lyctines have reported sex ratios approximately equal to 1 with females slightly outnumbered males in sapwood pieces [21,22,23] while on artificial diets, the sex ratio of *L. africanus* and *L. brunneus* were equal to 1 [16,24]. According to those data, the female survival rate from larva to adult might be higher than the male survival rate on the diets used in this study.

The body weight of the *L. africanus* fed the three different diets were similar. When rearing *L. brunneus* on sawdust- and cellulose-based diets (equivalent to Diet 1 and Diet 2 in this study), Iwata and Nishimoto [16] used body length as one of the parameters for body weight, and reported that the beetles reared on sawdust- and cellulose-based diets produced similar, well-developed (large) individuals. They also mentioned that there were no significant differences in body length among individuals reared on each diet. The present results suggest that Diet 1, Diet 2 and Diet 3 did not affect the food consumption of larval-stage *L. africanus*. In the case of other insects fed on low-quality foods, insects compensated for inadequate nutrient uptake by eating more food [25].

Based on our results, we concluded that the filler used in artificial diets for *L. africanus* seems to affect diet performance, which influenced the adult emergence. The amount of starch in the diet is not the only important factor to be considered when selecting a suitable diet for *L. africanus*. The filler should enhance oviposition and larval development [26]. In addition, the density of the artificial diet is an important factor influencing lyctines’ growth [19]. Utilization of alpha-cellulose as a filler of artificial diet for rearing *Lyctus* is not proposed due to its fiber characteristics.

Diet 1 and Diet 2 diets could be used for mass rearing *L. brunneus* [16]. However, it was reported that malformations were occasionally found in the abdominal segmentation of beetles that emerged from diets without sawdust [16]. These malformations were likely generated by the absence of some necessary compounds in cellulose-based diets [16], such as linoleic acid and sterols [27], steroid and minerals that are present in wood [28]. It was also reported that the cellulose-based diet (Diet 2) resulted in a higher number of malformed individuals than the wood-based (Diet 1) diet in *L. brunneus* [16]*.* Further study is needed to verify the malformation effects on *Lyctus* development. We conclude that Diet 2 could be utilized for mass culturing of *L. africanus* when sawdust or wood particles are not available.
